# The effects of castration (ram lambs versus wether lambs) on animal growth, feed efficiency, carcass characteristics, and cutability

**DOI:** 10.1093/tas/txaf154

**Published:** 2025-11-24

**Authors:** Kayla G Scott, Benjamin M Bohrer, Alejandro E Relling, Braden J Campbell

**Affiliations:** Department of Animal Sciences, The Ohio State University, Wooster, OH, 44691, United States; Department of Animal Sciences, The Ohio State University, Columbus, OH, 43210, United States; Department of Animal Sciences, The Ohio State University, Wooster, OH, 44691, United States; Department of Animal Sciences, The Ohio State University, Wooster, OH, 44691, United States

**Keywords:** carcass traits, gain to feed ratio, lamb production, meat yield

## Abstract

The aim of this experiment was to investigate the differences between intact (ram) and castrated (wether) lambs by evaluating animal growth pre- and post-weaning, feed efficiency, carcass characteristics, and carcass cutability. A total of 144 lambs were used for this feeding experiment (ram; n = 72 and wether; n = 72) and 70 lambs (ram; n = 35 and wether; n = 35) were slaughtered at the end of an 8-wk finishing period. Treatment (ram or wether) was randomly assigned shortly after birth. During the finishing period, lambs were weighed every 14 d. Lambs were slaughtered over five harvest dates based on live weight targets, evaluated for carcass characteristics 24-h postmortem, and fabricated into subprimal cuts 48-h postmortem, with weights collected from each subprimal. Results for the finishing period were analyzed using PROC MIXED in SAS with a fixed effect of sex with random effects of group (location and lambing season) and block. Carcass and economic value data were analyzed using PROC GLIMMIX in SAS with a fixed effect of sex and random effects of pen, block, and season. In terms of animal growth, there was a sex × time interaction (*P *< 0.01) for lamb body weight (BW), resulting in rams having heavier BW throughout the 8-wk finishing period. In addition, ram lambs also had a greater gain to feed (G:F) ratio (*P *< 0.01) and average daily gain (ADG) (*P *< 0.01). Furthermore, there was a sex × time interaction for dry matter intake (DMI) (*P *= 0.04) as ram lambs consumed more feed when compared with wether lambs. In the abattoir, ram lambs retained heavier carcasses (*P *< 0.01), with larger ribeyes (*P *< 0.01) and were trimmer (backfat thickness; *P *< 0.01 and bodywall thickness; *P *= 0.02), with overall lamb quality being equivalent between treatments as indicated by similar overall quality grades (*P *= 0.99) and degree of fat flank streakings (*P *= 0.53). Moreover, ram lambs were higher yielding for several meat cuts including neck, shoulder, foreshank, breast, trotters, and leg (*P *< 0.05) when compared with wether lambs. Within the context of this experiment, ram lambs exhibited greater weight gain, feed efficiency, carcass yield, and cutability. With uncertainties remaining as to the consumer acceptability of meat produced from ram lambs, coupled with increased DMI required by growing ram lambs; further investigation is needed to support the production of ram lambs for meat production.

## Teaser

Ram lambs had heavier final body weights and higher yielding carcasses when compared with wether lambs.

## Introduction

Research efforts investigating the effects of sex in lamb production in the United States have been an underrepresented area of research. In part, this is because sheep make up a small percentage of livestock inventories in both the United States as well as Ohio [approximately 3.1% of total livestock inventory (all beef cattle, hogs and pigs, sheep and lambs, goats); according to the United State Department of Agriculture inventory reports as of 1 January 2018] (USDA APHIS 2017). Given the potential to decrease management inputs and health concerns associated with castration (Schoenian 2007; Larrondo et al. 2018), in addition to increasing the marketability of intact or unadulterated lambs for ethnic religious celebrations (Bhatti et al. 2019), ram lambs may be more appealing to aid sheep producers in securing increased profits within their respective livestock enterprise.

Previous literature has evaluated the differences between lamb sex which analyzed live animal performance as well as carcass characteristics (Claffey et al. 2018a; Gravador et al. 2018; Schoenian et al. 2020). A multi-year study conducted at the University of Maryland investigating the use of East Friesian × Lacaune crossbred lambs found that ram lambs had heavier hot and cold carcass weights, larger ribeye areas, trimmer carcasses, and a greater percentage of boneless closely trimmed retail cuts (BCTRC) while wether lambs had greater dressing percentages (Schoenian et al. 2020). In Claffey et al. (2018a), the authors found that between rams and wethers across two different breeds (Scottish Blackface and Texel), rams were heavier at slaughter, had a greater average daily gain (ADG), greater feed conversion ratio (FCR), less fat over the loin muscles, and lesser dressing percentages; however, there were no differences in dry matter intake observed between sexes. Similarly, Gravador et al. (2018) observed crossbred (Texel × Scottish Blackface) lambs confirmed some of the results of Claffey et al. (2018a) reporting that rams had greater overall weight gain, ADG, and FCR in addition to less fat over the entire carcass.

While research efforts on the effects of sex in lamb production have provided valuable insights (Claffey et al. 2018a; Gravador et al. 2018; Schoenian et al. 2020), knowledge gaps still exist, especially in the context of sheep production in the Midwest and Eastern regions of the United States that rear early maturing breeds (*e.g.*, Dorset and Hampshire). As a result, this experiment aims to identify differences between ram and wether lambs as it relates to early maturing sheep breeds that are commonly raised in the eastern United States by evaluating live animal growth efficiency, carcass characteristics, and carcass cutability. The hypothesis is that ram lambs will produce heavier live weights and carcasses, that are trimmer, yielding heavier subprimal cuts without sacrificing carcass quality components, resulting in carcasses with greater economic value.

## Materials and methods

Approval of the protocol for this experiment was provided by The Ohio State University Animal Care and Use Committee (#2023A00000091). Animal care and management was in compliance with the United States Animal Welfare Act and the Guide for the Care and Use of Agricultural Animals in Agricultural Research and Teaching (FASS 2010).

### Experimental design

This experiment had two phases of data collection and measurement. The first of which aimed to evaluate live lamb performance metrics including body weight (BW) gain, feed efficiency represented as gain to feed (G:F) ratio, average daily gain (ADG), and dry matter intake (DMI). The second phase evaluated carcass characteristics and cutability. All lambs (n = 144) were utilized for the first phase and were managed at either The Ohio State University Small Ruminant Research Center (SRRC) in Wooster, Ohio (n = 70) or the Eastern Agricultural Research Station (EARS) in Caldwell, Ohio (n = 74). Lambs from the SRRC flock were crossbred (Dorset and Dorset × Hampshire × Ile de France) genetics and lambs from the EARS flock were primarily Dorset. Lambs that were utilized for the second phase of data collection were sourced from the SRRC flock with 70 lambs (ram; n = 35 and wether; n = 35) being used for carcass evaluation and cutability. Treatments of intact males (rams) and castrates (wethers) were randomly assigned to lambs shortly after birth. All lambs were weaned at approximately 60 d of age. Lambs were blocked by birth weight and sex and were randomly assigned to 36 pens with 3 lambs per pen at SRRC and 5-6 lambs per pen at EARS.

### Animal and nutrition management

The experiment consisted of two lambing periods: fall-born lambs (FBL) and spring-born lambs (SBL). Lambs were weighed at birth and castrated within 14 d of age in accordance with farm Standard Operating Procedures (SOP). All lambs remained with their respective dams until they were weaned. Lambs were weaned at approximately 60 d of age with lamb body weights being collected. Lambs were housed in an indoor feedlot, with pen sizes being approximately 3.6 x 1.6 m at SRRC and 6.0 × 1.8 m at EARS. After weaning and before the start of the finishing period, lambs were transitioned from the starter/creep feed ration (Diet 1; Table 1) to the finishing ration used for the 8-wk finishing period (Diet 2 and Diet 3; Table 1), over a period of approximately 4 wk. The same ration (Diet 1) was used in both lambing periods to transition lambs from the post-weaning period to the start of the finishing period; however, the FBL group received Diet 2 and the SBL group received Diet 3 during the duration of the 8-wk finishing period. Rations were approximately equivalent in nutritive quality and formulated to meet or exceed dietary nutrient requirements of growing lambs (NRC 2007). During the transition period, a biopsy sample of adipose and muscle tissues was collected from the longissimus lumborum muscle on the right side of the animal. Animals were randomly selected for biopsy, with 24 (rams; n = 12 and wethers; n = 12) samples taken in total. These samples were frozen for future analyses. At the initiation of the finishing period, lambs were weighed and sorted into treatment groups (ram or wether) and fed for 8 wk using the finishing rations described above. Lambs were offered approximately 3.25% of their BW as fed. During the finishing period, if no refusals were collected for two consecutive days, each pen’s daily feed allotment was increased by 10% of what was offered the previous day. Feed refusals were collected and weighed each day before new feed was offered. Water was provided *ad libtium.* Throughout the duration of the finishing period, lambs were weighed every 14 d until each group’s slaughter period, with lambs being slaughtered 55 d after the initiation of the finishing period.

**Table 1.** Diet composition and nutrient analysis reported on dry matter basis for ram and wether lamb diets offered during the transition and finishing phases.

Item	Diet 1	Diet 2	Diet 3
**Ingredient**	% dry matter basis		
**Corn (rolled and ground)**	52.1	61.1	61.1
**Alfalfa meal, dehydrated 16%**	30.0	…	…
**Soyhulls**	…	24.1	25.0
**Soybean Meal, 47% CP**	14.5	11.1	7.5
**Wheat middlings**	…	…	2.5
**Energ II^a^**	…	1.5	1.5
**Dicalcium phosphate**	1.1	…	…
**Limestone**	0.5	0.9	0.9
**Ammonium chloride**	…	0.4	0.5
**TM Salt^b^**	0.5	0.4	0.4
**Urea (prilled), 281% CP**	0.5	0.4	0.4
**Copper sulfate**	0.5	…	…
**Selenium, 201 ppm**	0.1	0.09	0.09
**Zinc oxide**	0.01	…	…
**Vitamin E, 44 IU/g**	0.09	0.04	0.04
**Vitamin D, 3000 IU/g**	0.02	0.01	0.001
**Vitamin A, 30000 IU/g**	0.01	0.01	0.001
**Bovatec 91, 200.6 g/kg**	0.01	0.01	0.02
**Analyzed nutrient content^c^, %**			
**CP^d^**	18.50	16.71	14.46
**NDF^d^**	21.00	17.15	24.94
**ADF^d^**	15.00	11.11	15.13

a Ca salts of fatty acids.

b Contained > 94.0% NaCl, 0.350% Zn, 0.20% Fe, 0.20% Mn, 0.030% Cu, 0.005% Co, 0.007% I.

c Feed samples were analyzed by Rock River Laboratory, Inc.

d Crude Protein (CP); Neutral Detergent Fiber (NDF); Acid Detergent Fiber (ADF).

### Carcass evaluation

Lamb live weight was collected immediately prior to slaughter at The Ohio State University Meat Laboratory in Columbus, Ohio. During the slaughter process, head weights were collected, with each head being removed flush with the posterior occipital bone of the skull. Additionally, testes were removed and weighed for each ram carcass, severing at the junction of the teste and spermatic cord. After carcasses were sprayed with water to remove contaminants, but before organic acids were sprayed for antimicrobial intervention, a biopsy of the adipose and muscle tissues was collected from the longissimus lumborum of the right side of the carcass, immediately caudal of the 13^th^ rib (scar tissue was used to identify the preceding biopsy collection site). These biopsy samples were frozen and stored for future analyses. Carcass characteristics were collected at 24-h postmortem. Carcasses were ribbed between the 12^th^/13^th^ rib interface to collect ribeye area, back fat depth, and body wall thickness measurements. The ribeye area was measured using an official United States Department of Agriculture (USDA) pork/lamb ribeye evaluation dot grid (Plastic Grid for Quick Measurement of Loin Eye, Iowa State University Extension and Outreach, Ames, IA, USA). Ribeye area was measured and collected in square inches, to determine the surface area of the longissimus muscle. The measurement for backfat thickness was collected from the center of the outer edge of each ribeye and the average thickness of the two measurements was recorded and utilized as final measure to reduce effects of human error during pelt removal. Body wall thickness was measured 12.7 cm diagonally from the vertebrae of each carcass and did not take into account the width of the rib. Additionally, leg muscle score (Smith et al. 2012) and overall carcass conformation (Greiner 2005) were evaluated to estimate muscle volume as well as fat distribution over the entire carcass, to determine overall quality grade (USDA AMS Livestock and Seed Division 1992). Similarly, fat flank streakings were evaluated by examining the fat accumulation on the interior flanks of the carcass to determine the quality grade of each carcass. In order to maintain consistency with these measures, the same trained individual evaluated each carcass for all attributes during the duration of this experiment. Dressing percentages were calculated by dividing the hot carcass weight by the live weight before multiplying the quotient by 100. Yield grade was calculated based upon the following formula: yield grade (YG) = [(backfat thickness (in) × 10) + 0.4]. The percentage of boneless closely trimmed retail cuts (BCTRC) was estimated by utilizing the following formula: BCTRC = 49.94 − {[0.085 × Hot Carcass Weight, lbs. (HCW)] − [4.38 × Back Fat, in. (BF)] − [3.53 × Body Weight, lbs. (BW)] + [2.46 × Ribeye Area, in.^2^ (REA)]}. Both the YG and BCTRC formulas were utilized based upon the guidelines from USDA AMS Livestock and Seed Division (1992).

### Carcass cutability

After 48 h postmortem, lamb carcasses were separated into hind saddle and fore saddle. Each carcass was then fabricated into primal and subprimal cuts following the USDA Institutional Meat Purchase Specifications (IMPS) guidelines (USDA IMPS 2022). Weights were collected from the leg (IMPS 233B), sirloin (IMPS 245), loin (IMPS 232A), loin flank (IMPS 232E), shoulder (IMPS 207), rack (IMPS 204A), foreshank (IMPS 210), hind shank (IMPS 233F), and breast (IMPS 209). Neck, plate, and trotters do not have specific IMPS and therefore are not recorded. To account for variation in final carcass weights, subprimal cut weights of each individual carcass were divided by the respective hot carcass weight to estimate a percentage that each cut represented.

### Economics and marketability

Live lamb value estimates were calculated by averaging the five sale dates (19 and 27 March 2024; 3 April 2024; 6 and 13 November 2024) from the Mt. Hope Auction report in Mt. Hope, Ohio. The value used to estimate lamb price was that which most closely represented the composition and grade of lambs in this experiment, Choice quality lambs that were 59 kg or heavier in weight. As livestock marketing prices from Mt. Hope are reported in hundredweight (cwt.), total price estimation was divided by 100 to calculate the price per pound. Estimated value of the total revenue from primal and subprimal cuts of each lamb carcass was calculated by averaging the values taken from the USDA Agricultural Marketing Service (AMS) Estimated National Lamb Carcass Cut-out value sheet. Those cuts that most closely represented the primal and subprimal cuts (leg, loin, flank, shoulder, rack, foreshank, breast, and neck) used in the cutability tests were used for the estimation. It should be noted that not all primal and subprimal cuts from this experiment are represented in the USDA AMS value sheet (*e.g.*, plate, sirloin, hind shanks, trotters) and therefore are not included in the cut-out estimate. The formula used to estimate lamb carcass cut-out value = [(Neck × 399.31) + (Rack × 1094.31) + (Shoulder × 406.73) + (Foreshank × 533.57) + (Breast × 393.60) + (Loin flank × 233.36) + (Loin × 930.63) + (Leg × 514.97)] × 2.2/100. Therefore, the estimated lamb carcass cut-out value calculated within the context of this experiment may not reflect the true or total value as it would in the industry given the lack of estimates provided for cuts without IMPS numbers and offal that would be generated from each carcass. Similarly, the estimated value of the carcass based upon the hot carcass weights of lambs in this experiment was calculated by using the gross carcass values from the USDA AMS carcass cut-out value sheet, whereas five values were averaged from each of the following 5 wk (21 and 28 March 2024; 4 April 2024; 7 and 14 November 2024) that lambs in this experiment were slaughtered.

### Statistical analysis

Data for both phases of the experiment were analyzed using a randomized complete block design. During the finishing phase, data were analyzed using PROC MIXED in SAS (SAS version 9.4, SAS Institute Inc., Cary, NC, USA) with fixed effects of sex and random effects of group (comprised of location and season) block. Blocking criteria were set by birth weight and treatment, and blocks were implemented on Day 1 of 8-wk finishing period. Birth weight and weaning weight data were analyzed with individual lamb as the experimental unit. For the 8-wk finishing period, pen was considered the experimental unit and in order to analyze the effect of treatment over time (sex × time interaction) repeated measures within pens with a covariate structure of compound symmetry was utilized. For lamb carcass metrics and economic value, data were analyzed using PROC GLIMMIX in SAS with fixed effects of sex and random effects of pen, block, and season. A *P* value of ≤ 0.05 was used to determine differences. The LSMEANS and PDIFF statements were used to determine estimates.

## Results

### Lamb live body weight and feed efficiency

As outlined in Table 2, lamb birth weight (*P *= 0.90) and weaning weight (*P *= 0.15) were not different between treatments. There was a sex × time interaction (*P *< 0.01) for lamb BW during the 8-wk finishing period whereas ram lambs had heavier BW when compared with wether lambs. Additionally, there was a sex × time interaction for lamb DMI (*P *= 0.04) during the 8-wk finishing period whereas ram lambs had a greater DMI when compared with wether lambs. There was no sex × time interaction observed for lamb ADG or G: F (*P *> 0.05). However, there was an effect of sex where ram lambs had a greater ADG (*P *< 0.01) and G:F (*P *< 0.01) when compared with wether lambs. Moreover, there was also an effect of time where ram lambs had a greater ADG (*P *= 0.03) and G:F (*P *< 0.01) when compared with wether lambs.

**Table 2.** Least squares means of live animal and feed efficiency data of ram and wether lambs from birth through the finishing phase of production.

Parameter	Ram	Wether	SEM^a^	*P* values		
				Sex	Time	Sex × time
**Birth weight, kg**	4.84	4.83	0.71	0.90	.	.
**Weaning weight, kg**	24.84	23.84	1.61	0.15	.	.
**8-wk finishing period body weights**						
**Start weight^b^, kg**	25.63	24.06	…	…	…	…
**Body weight, 14d, kg**	31.96^c^	28.83^d^	…	…	…	…
**Body weight, 28d, kg**	37.49^e^	33.72^f^	1.97	<0.01	<0.01	<0.01
**Body weight, 42d, kg**	43.87^e^	38.39^f^	…	…	…	…
**Final weight^b^, kg**	48.35^e^	41.99^f^	…	…	…	…
**8-wk finishing period feed efficiency**						
**DMI^g^, 0-14d, kg**	1.474	1.449	0.21	<0.01	<0.01	0.04
**DMI^g^, 15-28d, kg**	1.699	1.642				
**DMI^g^, 29-42d, kg**	1.969^e^	1.831^f^				
**DMI^g^, 43-56d, kg**	2.149^e^	1.930^f^				
**ADG^h^, kg**	0.394	0.324	0.02	<0.01	0.03	0.29
**G: F^i^, kg**	0.232	0.203	0.03	<0.01	<0.01	0.75

a SEM = standard error of the mean.

b Start weight indicates weight of lambs at the beginning of 8-wk finishing period and is considered Day 0. Final weight indicates weight of lambs at end of 8-wk finishing period and is considered Day 56.

c Differing subscript within row differ at a *P* value of <0.10.

d Differing subscript within row differ at a *P* value of <0.10.

e Differing subscript within row differ at a *P* value of <0.05.

f Differing subscript within row differ at a *P* value of <0.05.

g Average daily gain (ADG) calculated by dividing dry matter intake by days on feed.

h Dry matter intake (DMI).

i Gain to feed ratio (G:F) was calculated by dividing the amount gained by the DMI.

### Carcass characteristics

As shown in Table 3, ram lambs had heavier (*P *< 0.01) HCW, when compared with wether lambs. Additionally, head weights were heavier (*P *< 0.01) for ram lambs when compared with wether lambs. However, carcass dressing percentages were greater (*P *< 0.01) for wether lambs when compared with ram lambs. In terms of muscularity, ribeye area was larger (*P *< 0.01) for ram lambs when compared with wether lambs. Furthermore, ram lambs had less body fat when compared with wether lambs as shown by less back fat (*P *< 0.01) and a trimmer body wall measurement (*P *= 0.02). Of note, conformation score, leg score, fat flank streakings, and quality grade did not differ (*P *> 0.05) between treatments, with both groups achieving USDA Choice grades for conformation score, fat flank streakings, and quality grade. When considering YG, there was a difference (*P *< 0.01) between treatments, whereas ram lambs graded USDA YG 1 while wether lambs graded USDA YG 3. Moreover, the percentage of BCTRC was greater (*P *< 0.01) for ram lambs when compared with wether lambs.

**Table 3.** Least squares means of carcass weight and carcass characteristics of ram and wether lambs.

Parameter	Ram	Wether	SEM^a^	*P* value
**Hot carcass weight, kg**	36.95	34.29	2.84	< 0.01
**Testes weight^b^, kg**	0.61	.	0.06	.
**Head weight, kg**	2.81	2.38	0.13	<0.01
**Dressing percentage, %**	57.61	59.79	0.33	<0.01
**Ribeye area, cm²**	22.65	20.32	0.40	<0.01
**Backfat thickness, cm**	0.36	0.72	0.17	<0.01
**Body wall thickness, cm**	1.86	2.09	0.15	0.02
**Conformation score^c^**	5.45	5.21	0.51	0.45
**Leg Score^d^**	12.45	12.07	0.20	0.18
**Fat flank streakings^e^**	3.15	3.28	0.21	0.53
**Yield grade^f^**	1.83	3.23	0.66	<0.01
**Quality grade^g^**	2.14	2.14	0.08	0.99
**BCTRC^h^, %**	48.45	47.13	1.01	<0.01

a SEM = standard error of the mean.

b Testes weight collected from ram lambs (n = 35).

c Conformation score ranged from Good to Prime and utilized −, O, + systems. For data analysis purposes, Good− = 1, GoodO = 2, Good+ = 3, Choice− = 4, ChoiceO = 5, Choice+ = 6, Prime− = 7, PrimeO = 8, and Prime+ = 9.

d Leg score based on 9 (very thin muscling) to 15 (very thick muscling) scoring system.

e Fat flank streakings ranged from Traces to Moderate. For data analysis purposes, Traces = 2, Slight = 3, Small = 4, Modest = 5, and Moderate = 6.

f Quality grade ranged from Good to Prime. For data analysis purposes, Good = 1, Choice = 2, and Prime = 3.

g Yield grade was calculated based upon the following formula: Yield Grade = [(backfat thickness, in. × 10) + 0.4].

h BCTRC = boneless closely trimmed retail cuts and calculated using the following formula: BCTRC, % = [49.94 − (0.085 × hot carcass weight, lb.) − (4.38 × backfat thickness, in.) − (3.53 × body wall thickness, in.) + (2.46 × ribeye area, in.^2^)].

### Carcass cutability

In Table 4, neck and trotter weights (*P *< 0.01) as well as neck and trotter weights when represented as a percentage of HCW (*P *< 0.01, *P *= 0.04; respectively) were greater for ram lambs when compared with wether lambs. Moreover, ram lambs had heavier shoulder (*P *< 0.01), breast (*P *= 0.01), foreshank (*P *= 0.03) and leg weights (*P *< 0.01) when compared with wether lambs. Rack, plate, hindshank, loin, and sirloin weights as well as rack, plate, shoulder, foreshank, breast, hindshank, loin, leg, sirloin weights as a percentage of HCW, did not differ (*P *> 0.05) between treatments.

**Table 4.** Least squares means of carcass cutability of ram and wether lambs.

Parameter^a^	Ram	Wether	SEM	*P* values
**Neck, kg**	1.44	1.22	0.04	<0.01
** Neck percentage of HCW, %**	3.93	3.57	0.28	<0.01
**Rack, kg**	2.80	2.64	0.39	0.39
** Rack percentage of HCW, %**	7.57	7.65	0.48	0.85
**Plate, kg**	1.43	1.38	0.13	0.49
** Plate percentage of HCW, %**	3.85	4.00	0.11	0.35
**Shoulder, kg**	7.03	6.40	0.39	<0.01
** Shoulder percentage of HCW, %**	19.00	18.64	0.44	0.43
**Foreshank, kg**	2.05	1.86	0.16	0.03
** Foreshank percentage of HCW, %**	5.55	5.43	0.22	0.65
**Breast, kg**	1.92	1.71	0.29	0.01
** Breast percentage of HCW, %**	5.13	4.96	0.43	0.42
**Hindshank, kg**	1.14	1.14	0.04	0.92
** Hindshank percentage of HCW, %**	3.13	3.31	0.23	0.30
**Trotters, kg**	1.05	0.92	0.02	<0.01
** Trotters percentage of HCW, %**	2.84	2.70	0.20	0.04
**Loin flank, kg**	1.46	1.60	0.46	0.46
** Loin flank percentage of HCW, %**	3.88	4.68	1.01	0.20
**Loin, kg**	3.50	3.45	0.21	0.69
** Loin percentage of HCW, %**	9.40	9.94	0.25	0.14
**Leg, kg**	6.93	6.07	0.41	<0.01
** Leg percentage of HCW, %**	18.68	17.63	0.67	0.07
**Sirloin, kg**	3.57	3.37	0.31	0.10
** Sirloin percentage of HCW, %**	9.68	9.82	0.18	0.59

a Institutional Meat Purchase Specifications (IMPS) guidelines (2014) were used to specify the primal and subprimal cuts collected. IMPS numbers and corresponding cuts: leg (IMPS 233B), sirloin (IMPS 245), loin (IMPS 232A), loin flank (IMPS 232E), shoulder (IMPS 207), rack (IMPS 204A), breast (IMPS 209) foreshank (IMPS 210) and hind shank (IMPS 233F). Neck, plate, and trotters do not have specific IMPS.

### Economics and marketability

As outlined in Table 5, ram lamb carcasses were found to be of greater value with greater estimated revenue for marketing based upon live animal weight (Live value) (*P *< 0.001), hot carcass weight (Gross carcass value) (*P *< 0.001), and primal and subprimal cuts (Cut-out value) (*P *= 0.001) when compared with wether lamb carcasses.

**Table 5.** Estimated values (USD, $) of ram and wether lambs when marketing animals on a live weight (kg), hot carcass weight (kg), or cut-out value basis.

Parameter	Ram	Wether	SEM^a^	*P* values
**Live value^b^**	263.16	235.03	20.53	<0.001
**Gross carcass value^c^ ^,^ ^d^**	446.58	414.46	34.32	0.001
**Cut-out value^c^ ^,^ ^e^**	341.14	316.60	27.79	<0.001

a SEM = standard error of the mean.

b Live value estimated based upon local (Mt. Hope Auction, Mt. Hope, Ohio) live market values representative of the lambs used in this experiment. Averages from dates closest to slaughter dates [19 March 2024 (https://mthopeauction.com/wp-content/uploads/LMR-Market-Report3-19-24.pdf), 27 March 2024 (https://mthopeauction.com/wp-content/uploads/LMR-Market-Report3-27-24.pdf) 3 April 2024 (https://mthopeauction.com/wp-content/uploads/LMR-Market-Report4-3-24.pdf), 6 November 2024 (https://mthopeauction.com/wp-content/uploads/LMR-Market-Report11-6-24.pdf), 13 November 2024 (https://mthopeauction.com/wp-content/uploads/LMR-Market-Report11-13-24.pdf)] for Choice lambs 130 pounds or greater were used to estimate value.

c Gross carcass value and carcass cut-out value estimated based upon USDA Agricultural Marketing Service Estimated National Lamb Carcass Cut-out values from each of the weeks of the five slaughter dates and averaged: 21 March 2024 (https://mymarketnews.ams.usda.gov/filerepo/sites/default/files/2649/2024-03-21/1104612/ams_2649_01038.pdf), 28 March 2024 (https://mymarketnews.ams.usda.gov/filerepo/sites/default/files/2649/2024-03-28/1106622/ams_2649_01043.pdf), 4 April 2024 (https://mymarketnews.ams.usda.gov/filerepo/sites/default/files/2649/2024-04-04/1108541/ams_2649_01048.pdf), 7 November 2024 (https://mymarketnews.ams.usda.gov/filerepo/sites/default/files/2649/2024-11-07/1169942/ams_2649_01200.pdf), and 14 November 2024 (https://mymarketnews.ams.usda.gov/filerepo/sites/default/files/2649/2024-11-14/1171792/ams_2649_01205.pdf).

d The following formula was used to estimate Gross carcass value: HCW value = HCW × 549.33 × 2.2/100.

e Values of primal and subprimal cuts from USDA AMS report that most closely represent the corresponding primal and subprimal cuts from this experiment (Only those cuts represented in the USDA AMS report that were replicated in this experiment were used. As a result, some cuts were not represented in the formula.) were utilized for the estimation. The following formula was used to estimate the cut-out value: Cut-out value = [(Neck × 399.31) + (Rack × 1094.31) + (Shoulder × 406.73) + (Foreshank × 533.57) + (Breast × 393.60) + (Loin flank × 233.36) + (Loin × 930.63) + (Leg × 514.97)] × 2.2/100.

## Discussion

While nuances in differences between ram and wether lambs have been widely recognized by sheep producers, quantifying those differences has not been a popular investigation. In this experiment, ram and wether lambs were evaluated for live animal growth and feed efficiency, carcass characteristics and cutability, and estimation of potential revenue. Within this experiment, it was hypothesized that ram lambs would produce heavier live animals and carcasses, that are trimmer throughout the length of the carcass, without sacrificing carcass quality characteristics. By keeping ram lambs intact, labor inputs involved with castration could be reduced, saving producers time and resources. Furthermore, some American lamb marketing channels, unadulterated or unblemished animals are demanded and thus the rearing and marketing of ram lambs may be implemented into a farm management plan as a niche marketing strategy.

Within this experiment, it was found that ram lambs were heavier throughout the 8-wk finishing period, with a 6% difference in body weight at the start of the finishing period and a 13% difference at the end of the finishing period when compared with wether lambs. Additionally, throughout the duration of the finishing period, ram lambs had a greater DMI while demonstrating nearly 18% greater ADG and 12.5% greater G: F when compared with wether lambs. This difference in DMI intake at the start of the finishing period was approximately 1.5% and grew to a 10% difference by the end of the finishing period. These differences may be attributed to ram lambs requiring greater feed intake to support the rapid muscle growth via increased protein synthesis. This increase in lean tissue accumulation is supported by increased concentrations of testosterone in ram lambs (Schanbacher and Ford 1976; Schanbacher 1980; Unruh 1986), as this anabolic steroid hormone suppresses adipogenesis (Puttabyatappa et al. 2018) and promotes muscle protein synthesis directly by promoting anabolism (Schanbacher et al. 1980) or through increasing concentrations of growth hormones, such as insulin-like growth factor-I (Arnold et al. 1996).

Increases in lamb growth corroborate those reported by Claffey et al. (2018a) and Gravador et al. (2018) as these authors reported heavier final BW at the time of slaughter for ram lambs when compared with wether lambs. However, Claffey et al. (2018a) reported no differences between sexes (rams versus wethers) in terms of DMI, conflicting with the results from this experiment that resulted in an over 0.2 kg difference between treatments at the end of the 8-wk finishing period. Differences in lamb DMI between the current experiment and Claffey et al. (2018a) could be partially explained by differences in diets offered. In the current experiment, no forage was offered with the concentrate feed, whereas the diet provided by Claffey et al. (2018a) consisted of both concentrates and forages. Offering diets that are greater in fiber concentration will decrease DMI (Claffey et al. 2018b). Therefore, assessing this relationship of DMI relative to the rate at which ram lambs achieve marketable weights is critical for the implementation of this management strategy given that feed costs represent a substantial expense. In a similar experiment, Jaborek et al. (2018) evaluated live animal and carcass traits of long-fed (218 d on feed) wether and ewe lambs. It was found that wether lambs were unable to offset the greater DMI with a greater amount of gain [feed cost per gain, $/kg (*P *> 0.05)] when compared with ewe lambs. These findings further support the need for this metric to be evaluated when comparing ram and wether lambs.

While investigating live animal production and efficiency of ram lambs, Seoni et al. (2018) completed an experiment that evaluated the effects of dietary protein and the presence of tannins in ram lamb finishing diets. The lambs in Seoni et al. (2018) were of similar size and maturity (approximately 3 mo of age) to those evaluated in the current experiment prior to the 8-wk finishing period, during the weaning and transitioning phases of production. In Seoni et al. (2018), ram lamb growth performance mirrored that of those ram lambs in the current experiment. As expected, ram lambs from the current experiment had a greater feed intake throughout the 8-wk finishing period, as the lambs remained on feed for additional period of time; however, ram lambs from this experiment were also able to achieve a greater ADG when compared with all four treatment groups from Seoni et al. (2018). These differences in animal weight are mainly attributed to animal age and days on feed prior to harvest.

While diet composition remains an important factor for live animal efficiency, it calls into question the ability of wether lambs to maintain similar growth and efficiency as ram lambs. Diets differing in quality or nutrient availability may aid increasing wether lamb growth. In Jaborek et al. (2018), the authors found that those lambs fed *ad libitum* whole shell corn (WSC) had greater ADG than those fed *ad libitum* alfalfa pellets or limit fed WSC. Similarly to Ellison et al. (2022), the lambs in Jaborek et al. (2018) fed WSC (both *ad libitum* and limit fed) or ‘concentrate’ diets consumed less feed than those that were forage-fed. In contrast, Ellison et al. (2022) found that wether lambs also had greater ADG, when forage-fed. While Ellison et al. (2022) did not observe any differences in carcass characteristics because of diet, Jaborek et al. (2018) found that lambs fed WSC had greater HCW, dressing percentages, and cost less to feed lambs to a harvest appropriate weight when compared with those lambs finished on a forage-based diet. Although diets did not differ in the current experiment, Ellison et al. (2022) and Jaborek et al. (2018) highlight the importance of investigating this concept further as diet may influence lamb growth and carcass quality.

Furthermore, while ram lambs had greater ribeye areas as well as less fat throughout the length of the carcass, it is understandable that ram lambs yielded a greater estimated percentage of BCTRC. Carcass BCTRC is an estimate of lean tissue yield and takes into consideration those factors related to muscling and trimness in addition to accounting for HCW. Both treatments in the current experiment had greater BCTRC percentages than those reported in Jaborek et al. (2018) and Ellison et al. (2022). Interestingly, lesser dressing percentages were found in this experiment when compared with Jaborek et al. (2018), but dressing percentages were still greater than those reported by Claffey et al. (2018a). The lambs in Jaborek et al. (2018) were considered long-fed lambs (218 d) and the extra days on feed increased the amount of muscle and fat deposited throughout the carcass, increasing the proportion of non-visceral weight on each carcass, in turn yielding greater dressing percentages.

Hot carcass weights and head weights were greater for ram lamb carcasses (Table 3) when compared with wether lamb carcasses in the current experiment. In the case of the current experiment, increased muscle and bone mass in ram lambs would increase carcass weight as both tissues remain with the carcass post-harvest. Increased bone mass is mainly attributed to an increased concentration of testosterone in intact males (Peralta et al. 1994; Quaresma et al. 2017). Furthermore, the increase in bone mass as well as the presence of testes resulted in a lesser dressing percentage for ram lambs when compared with wether lambs. While not commenting on the bone mass in meat cuts, the authors of Claffey et al. (2018a) attributed greater dressing percentages of wether lambs for both breeds evaluated (Texel-Scottish Blackface crosses and purebred Scottish Blackface) to increased offal of ram lambs, having larger, denser skulls in addition to the presence of testes. Additionally, rams were found to have a greater ribeye area, with less fat opposite the ribeye (backfat thickness), and less fat along the body wall. In Jaborek et al. (2018) investigating long-fed ewes and wethers, the authors found that wethers had equal ribeye areas (both approximately 22 cm^2^) to ram lambs in this experiment. However, in their paper, wethers spent a greater number of days on feed (218 total days on feed vs. 60 d for finishing period) to attain a similar result.

Carcass yield and subsequent predicated saleable product from ram lambs support the hypothesis that when marketed as a carcass or subprimal cuts, there is increased value for ram lambs within the context of this experiment as shown by an 11% increase in live animal value and a 7% increase in both gross carcass and cut-out values when compared with wether lambs. While Johnson et al. (2005) did not directly evaluate the value from subprimal cuts, but rather the overall carcass composition and muscle to bone ratios of ewe and ram lambs; these authors did find that most muscles (M. semimembranosus, M. semitendinosus, M. biceps femoris, and M. adductor) were larger and heavier for ram lambs than those from ewe lambs. Furthermore, Johnson et al. (2005) evaluated Texel-cross lambs for carcass characteristics and meat quality measures of pasture raised lambs of similar maturity to the lambs evaluated in the current experiment. Although an economic analysis evaluating the value of lamb carcasses was not conducted in Johnson et al. (2005), given a greater lean meat yield, it can be interpreted that ram lambs would produce carcasses of greater value. In the current experiment, when fabricating carcasses into wholesale cuts, neck, plate and trotter weights were greater for rams, which may be expected based on greater bone mass (Peralta et al. 1994; Quaresma et al. 2017) as these three cuts that have a considerable amount of bone.

## Conclusion

As outlined, ram lambs are more efficient as demonstrated by 13% heavier finishing weights coupled with a 12.5% greater G: F in addition to generating greater value when marketed on a live weight (11%), HCW (7%), or as boxed lamb cuts (7%) basis. However, it should be noted that this practice may not fit all production systems and further work should be investigated to better understand the broader consequences of implementing this strategy in a production setting and on a large scale. While the current experiment demonstrates that ram lambs were heavier, trimmer, and had greater feed efficiency, understanding the length of time required for ram lambs to achieve a marketable weight (in comparison to wether lambs) is of importance to better understand feed efficiency. Additional research investigating the effect of age and diet is warranted to identify the broader impacts of incorporating meat produced by intact males, especially as it relates to palatability and quality of meat products produced.
